# Predictors of survival among children with Burkitt lymphoma treated at a single center in Tanzania: a retrospective cohort study

**DOI:** 10.1016/j.htct.2026.106496

**Published:** 2026-07-06

**Authors:** Aika A. Shoo, Advera I. Ngazia, Nahya S. Masoud, Shakilu Jumanne, James J. Yahaya

**Affiliations:** a Department of Pathology, Muhimbili National Hospital, Dar-es-salaam, Tanzania; b Department of Pathology, School of Diagnostic Medicine, Muhimbili University of Health and Allied Sciences, Dar-es-salaam, Tanzania; c Department of Pediatric and Child Health, School of Medicine and Dentistry, University of Dodoma, Dodoma, Tanzania; d Department of Pathology, School of Health Sciences, Soroti University, Soroti, Uganda

**Keywords:** Burkitt lymphoma, Mortality, Predictors, Children

## Abstract

**Background:**

Despite improvements in the management of patients with Burkitt lymphoma, the survival rate in low- and middle-income countries remains poor. This study aimed to determine the predictors of 18-month overall survival among children with Burkitt lymphoma.

**Methods:**

A retrospective cohort study was conducted at the Pediatric Oncology unit of Muhimbili National Hospital in Dar-es-salaam, Tanzania. The study included a cohort of 123 children who were diagnosed with Burkitt lymphoma from January 2012 to December 2017. Predictors of mortality were assessed using Cox regression analysis. A two-tailed p-value <0.05 was considered statistically significant.

**Results:**

The median age of the patients was seven years and the majority 61.8% (76/123) were males. Abdominal presentation was the most common tumor site in 39% (48/123). The 18-month overall survival rate was 54.0%. Key predictors of mortality included human immunodeficiency virus (HIV) infection (adjusted Hazard ratio [aHR] = 5.12; 95% CI: 1.39–19.0; p < 0.01) and elevated lactate dehydrogenase levels (>500 U/L; aHR = 2.50; 95% CI: 1.14–14.71; p = 0.03).

**Conclusion:**

Most patients presented with abdominal Burkitt lymphoma. Despite the tumor's high chemosensitivity, treatment response in this cohort was marginal, characterized by moderate overall survival and low event-free survival. HIV infection and elevated lactate dehydrogenase levels were significant predictors of mortality. Improving outcomes will require optimized early diagnosis, HIV screening, and rigorous patient follow-up.

## Introduction

Burkitt lymphoma (BL) is a highly aggressive non-Hodgkin lymphoma (NHL) which was first described in 1958 by Denis P Burkitt in Kampala, Uganda [1]. His findings followed observations of an increased frequency of children with masses involving the face or abdomen [1,2]. BL is characterized by clonal proliferation of mature B-lymphocytes due to a chromosomal translocation t(8,14) that results in dysregulation of the c-MYC oncogene [3, 4, 5]. The World Health Organization (WHO) has classified BL into three distinct clinical variants: endemic, sporadic, and immunodeficiency-associated, the latter of which is predominantly observed in human immunodeficiency virus (HIV)-positive patients [6]. BL is the most common childhood cancer in equatorial Africa, accounting for 30%–50% of all childhood malignancies in children <15 years of age [2] and 90% of all childhood lymphoma [7]. The incidence of endemic BL in Tanzania, like in many sub-Sahara African countries, is difficult to ascertain due to the lack of a centralized population-based registry on childhood cancer [8].

Despite its aggressive nature, BL is highly responsive to chemotherapy. Some patients achieve complete remission with single-agent regimens, and relapse is rare beyond one year after treatment [9]. While cure rates for BL in resource-rich settings exceed 90%, most cancer treatment centers in sub-Sahara African report survival rates ranging between 25% and 62% [10]. Studies on reduced-intensity BL chemotherapy protocols have been adapted to suit the available resources and the level of supportive care in resource-limited settings [11, 12, 13]. Other studies have shown consistent and reproducible findings, leading to universally accepted protocols for use in these environments [14,15]. Factors hindering the development of resource-appropriate protocols in these settings include a lack of well-designed multicenter clinical trials for endemic BL, diverse sociocultural challenges, and comorbidities, such as HIV and severe malnutrition, that negatively impact treatment outcomes [11]. In addition to clinical variables, several socioeconomic factors unique to resource-limited settings significantly influence BL outcomes. Chief among these are diagnostic delays resulting from limited healthcare access, and the financial burden of care associated with low socioeconomic status. These systemic barriers frequently culminate in high rates of treatment abandonment, further compromising survival [10,16].

This study aims to review the clinical profile, survival, and predictors of mortality of children with BL treated in the Pediatric Oncology Unit (POU) at Muhimbili National Hospital (MNH) from January 2012 to December 2017.

## Methods

### Study design and setting

The POU was established in April 2011 after relocating from the Ocean Road Cancer Institute (ORCI), the country's primary cancer center. MNH is a national hospital and a teaching facility for the Muhimbili University of Health and Allied Sciences (MUHAS) in Dar es Salaam. The pediatric unit consists of two wards and a hostel providing both outpatient and inpatient services. With a 96-bed capacity, the unit averages 400 pediatric cancer admissions annually. Critically ill patients receiving chemotherapy or radiotherapy are admitted to the Upendo ward, while stable patients are cared for in the Tumaini ward. The hostel provides accommodation for pediatric patients from upcountry until their treatment is complete.

### Patients’ characteristics

All children aged less than 18 years who were diagnosed histologically with BL over a period of six years from January 2012 to December 2017 were included in this study if they had complete clinical information as well as follow-up information. Moreover, all patients who were included in the analysis were screened for HIV status during normal clinic attendance. Patients who died or abandoned care before the initiation of treatment, patients with a diagnosis of BL based on clinical suspicion, and those with missing information on treatment were all excluded from the analysis.

### Sample size and sampling procedure

The sample size was calculated using Kelsey method for cohort studies via OpenEpi [17] based on the following assumptions for comparing survival rates between early and advanced tumor stages: an 18-month survival rate of 51% for Stages I/II and 21% for Stages III/IV, based on previous research [10]. Additional parameters included a 95% confidence level (α = 0.05), a two-sided significance test, 90% power (β = 0.10), a 1:1 ratio between groups, and a 10% adjustment for loss to follow-up. A minimum sample size of 123 was deemed adequate to detect a 30% difference in survival rate. Sampling of the study subjects was based on the convenience sampling method, in which all participants meeting the inclusion criteria were consecutively enrolled until the sample size was obtained.

### Data collection

Data were extracted from patient files and electronic databases and recorded using a custom-designed data collection sheet. The extracted data included demographic (age and sex), clinical characteristics (tumor site, nutritional status, HIV status, chemotherapy treatment administered and response to treatment at discharge), and follow-up data (date of last visit and date of death).

### Institutional chemotherapy regimens for Burkitt lymphoma

Since 2011, children diagnosed with BL at MNH have been treated with the International Network for Cancer Treatment and Research (INCTR-03–06) protocol. The first-line regimen consists of a combination of cyclophosphamide, vincristine, and methotrexate (COM), supplemented by intrathecal methotrexate and cytarabine for central nervous system (CNS) prophylaxis or treatment [18]. Additionally, with this protocol, patients with refractory disease or early relapse were treated using a combination of etoposide, mesna, ifosfamide and cytarabine (EMIC) as a salvage regimen. Previous published data on the study using this BL treatment protocol reported overall survival (OS) rates of 67% and 62% at one and two years, respectively but there have been no follow-up review on the performance of this protocol of children treated using routine clinical patient care at MNH [19].

### Tumor staging

Patients in this study were staged using the St. Jude/Murphy staging system. The staging process included a clinical examination, a complete blood count, and an evaluation of peripheral blood films. Additionally, bilateral bone marrow aspirates and biopsies, cerebrospinal fluid (CSF) analysis, and imaging by computed tomography (CT) or magnetic resonance imaging (MRI), were performed for patients whose parents or guardians could afford them. Bone marrow involvement was defined as the presence of malignant lymphoma cells in a bone marrow smear [20]. Confirmation of CNS involvement was based on the presence of at least one of the following: lymphoma cells in the CSF cytology, intracranial or parameningeal lesions on imaging, cranial nerve palsy, or clinical spinal cord compression.

### Risk group classification

In this study, risk group classification was based on the INCTR-03–06 protocol in which patients were stratified as low-risk or high-risk similar to a previous study [19]. The low-risk group consisted of patients with a single extra-abdominal tumor the widest diameter of which was <10 cm. Furthermore, patients were classified as high-risk if they presented with features not seen in the low-risk group, such as advanced tumor stages (Stage III/IV), elevated lactate dehydrogenase (LDH) levels, intra-abdominal disease, a tumor diameter ≥10 cm, or involvement of the CNS or bone marrow.

### Tailoring of therapeutic regimen according to the patient risk group

Low-risk patients received three courses of cyclophosphamide, vincristine or oncovin, and methotrexate (COM) at three-week intervals. In contrast, high-risk patients received six courses of COM combined with intrathecal methotrexate and cytarabine at two-week intervals. With the exception of low-risk patients, treatment began with a pre-phase consisting of prednisone, followed by low-dose cyclophosphamide, vincristine, and prednisone (COP). Therapy was subsequently intensified for high-risk patients who failed to respond to COM, as well as for any patient with residual viable cells following the consolidation phase.

### Treatment response criteria

Treatment responses were categorized as follows: complete response (CR), defined as no evidence of disease post-treatment; partial response (PR), a reduction in tumor size of 50% or greater; no response (NR), characterized by persistent disease; and response not assessed (NRA) for patients who died or abandoned care prior to assessment [19]. Treatment response was assessed using clinical factors, including the Eastern Cooperative Oncology Group (ECOG) performance status, imaging (CT or MRI), and laboratory studies such as LDH levels. Tumor size reduction of ≥50% was evaluated after two weeks of chemotherapy. Evaluation methods included the monitoring of LDH levels, the clearance of lymphoma cells from the bone marrow, and radiographic assessment via CT or MRI.

### Measurement of variables

Wherever possible, the parents or guardians of patients who were diagnosed and treated from January 2012 to December 2017 were contacted by phone from January to December 2019. Caregivers who answered the phone were asked about the progress of the patients, whether they had died after treatment or relapsed. Additionally, patients were monitored at the last follow-up appointment visit in the facility. Patients who were unreachable by phone or who did not respond to call attempts were categorized as lost to follow-up. OS was defined as the time from the date of diagnosis or starting treatment to the date of death from any cause [21].

### Statistical analysis

Data were anonymized and recorded in the Epidata database version 2.0 and then exported to IBM SPSS version 20 for statistical analysis. Categorical variables are summarized as frequencies and percentages whereas continuous variables are reported as medians and interquartile ranges (IQR). The log-rank test was used to estimate the differences in survival groups. Cox regression analysis was performed to examine if any factors were associated with mortality. Factors associated with mortality in the univariate analysis with a p-value <0.2 were entered in the multivariate analysis to determine predictors of mortality. Kaplan-Meier curves were used to estimate the OS at 18 months since the start of treatment. A two-tailed p-value <0.05 was considered statistically significant.

## Results

**Figure 1:**
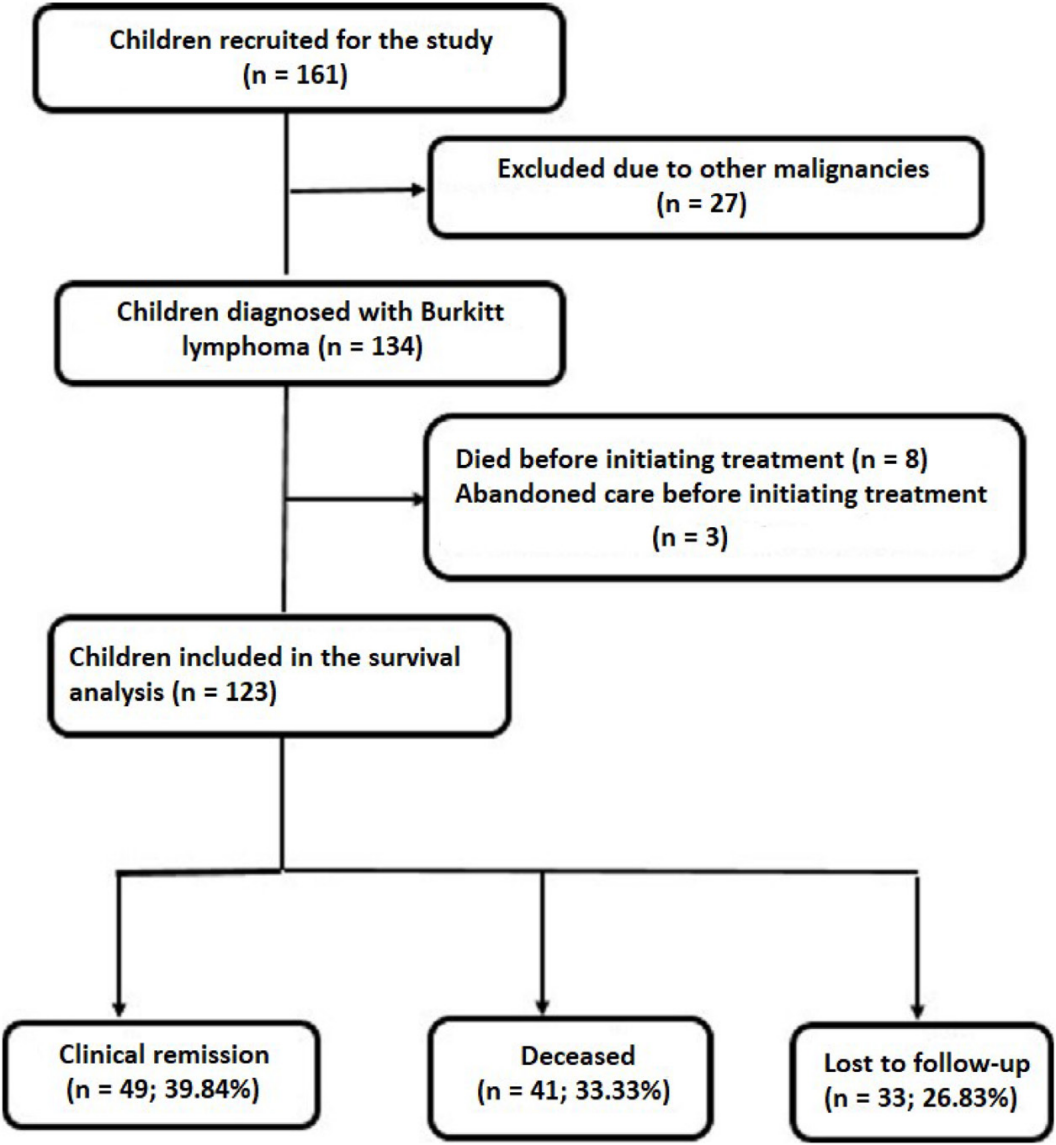
Flow chart showing enrolled study children and their outcome.

A total of 161 children clinically suspected of having BL were identified from the admissions register, 27 (16.8%) of whom were excluded after reviewing their case notes as they had other malignancies. Of the 83.2% (134/161) children that remained as cases of BL, 8.2% (11/134) were excluded due to death or abandonment of care before treatment initiation. A total of 91.8% (123/134) children were finally eligible to be included in the final analysis (Figure 1).

### Demographic and clinical characteristics of the patients

The median age at diagnosis was 7 (range: 4 −10) years, with a male to female ratio of 1.6:1. The majority (n = 102; 82.9%) presented with symptoms of less than three months duration. Abdominal involvement accounted for over one-third (n = 48; 39.0%) followed by jaw involvement found in 31 (25.2%) of the cases. Most of the patients (n = 69; 56.1%) had endemic BL. A CSF evaluation was performed in 97 (78.9%) children, among whom 39 (40.2%) had CNS involvement. A bone marrow evaluation was performed in 98 (79.7%) patients, of whom 12.2% (n = 12) had bone marrow involvement. Regarding risk group classification, the vast majority 69.9% (n = 86) of the patients were in the high-risk group. Among the 98 patients (79.7%) who underwent clinical staging, Stage IV was the most prevalent, accounting for 38.8% (n = 38/98) of the cohort, followed by Stage III in 28.6% (n = 28/98) of cases (Table 1).

**Table 1:** Demographic and clinical characteristics of children with Burkitt lymphoma (n = 123).

Variable	n		%
Sex			
Male	76		61.8
Female	47		38.2
Age at diagnosis (years)			
<10	41		33.3
≥10	82		66.7
Duration of illness (months)			
<3	102		82.9
≥3	21		17.1
Tumor sites			
Abdomen	48		39.0
Jaw	31		25.2
Abdomen and jaw	25		20.3
Others *	19		15.5
CSF testing			
Positive	39		31.7
Negative	58		47.2
Missing data	26		21.1
Bone marrow evaluation			
Positive	12		12.2
Negative	86		69.9
Missing data	25		20.3
Tumor staging			
Stage I	10		8.1
Stage II	22		17.9
Stage III	28		22.8
Stage IV	38		30.9
Missing data	25		20.3
Risk groups			
Low-risk	12		9.8
High-risk	86		69.9
Missing data	25		20.3
Subtypes of Burkitt lymphoma			
Endemic	69		56.1
Sporadic	46		37.4
HIV-associated	8		6.5
HIV status			
Positive	8		6.5
Negative	115		93.5
LDH (U/L)			
<250	3		4.1
250–500	16		21.9
>500	54		74.0
Hemoglobin (g/L)			
<70	17		13.8
70–99	27		22.0
100–110	57		46.3
>110	22		17.9

CSF: cerebrospinal fluid; LDH: lactate dehydrogenase.

⁎ Other tumor sites included orbit, ovary, neck, chest, femur, and spinal column.

### Treatment regimens of the patients and related complications

Most patients were treated with chemotherapy only (n = 51; 41.5%) followed by chemotherapy and surgery (n = 28; 22.8%). Over one-third (n = 48; 39.0%) of those who were treated with chemotherapy were prescribed the COM regimen. Most patients (n = 63; 51.2%) underwent 1–15 cycles of chemotherapy. Additionally, 21 patients (17.1%) had treatment-related complications (Table 2**).**

**Table 2:** Treatment regimens for the patients and related complications (n = 123).

Variable	N	%
Treatment modalities		
Chemotherapy	51	41.5
Chemotherapy + surgery	28	22.8
Chemotherapy + surgery + radiotherapy	19	15.4
Chemotherapy + radiotherapy	15	12.2
Chemotherapy + surgery + physiotherapy	10	8.1
Types of chemotherapy regimens given		
COM	48	39.0
R-COM	11	8.9
CHOP	23	18.7
ITmCH	9	7.3
EMIC	32	26.0
Number of cycles		
1–15	63	51.2
16–31	34	27.6
>31	26	21.2
Treatment related complications		
Nausea	9	7.3
Vomiting	4	3.3
Anaemia	5	4.1
Others	3	2.4
None	102	82.9

COM: cyclophosphamide + vincristine + methotrexate; R-COM: Rituximab + cyclophosphamide + vincristine + methotrexate; CHOP: cyclophosphamide + doxorubicin + vincristine + prednisolone; ITmCH: intrathecal methotrexate + cytarabine + hydrocortisone; EMIC: etoposide + mesna + ifosfamide + cytarabine.

#### Treatment responses of children with Burkitt lymphoma to first- and second-line regimens using the INCTR-03-06 protocol

**Figure 2:**
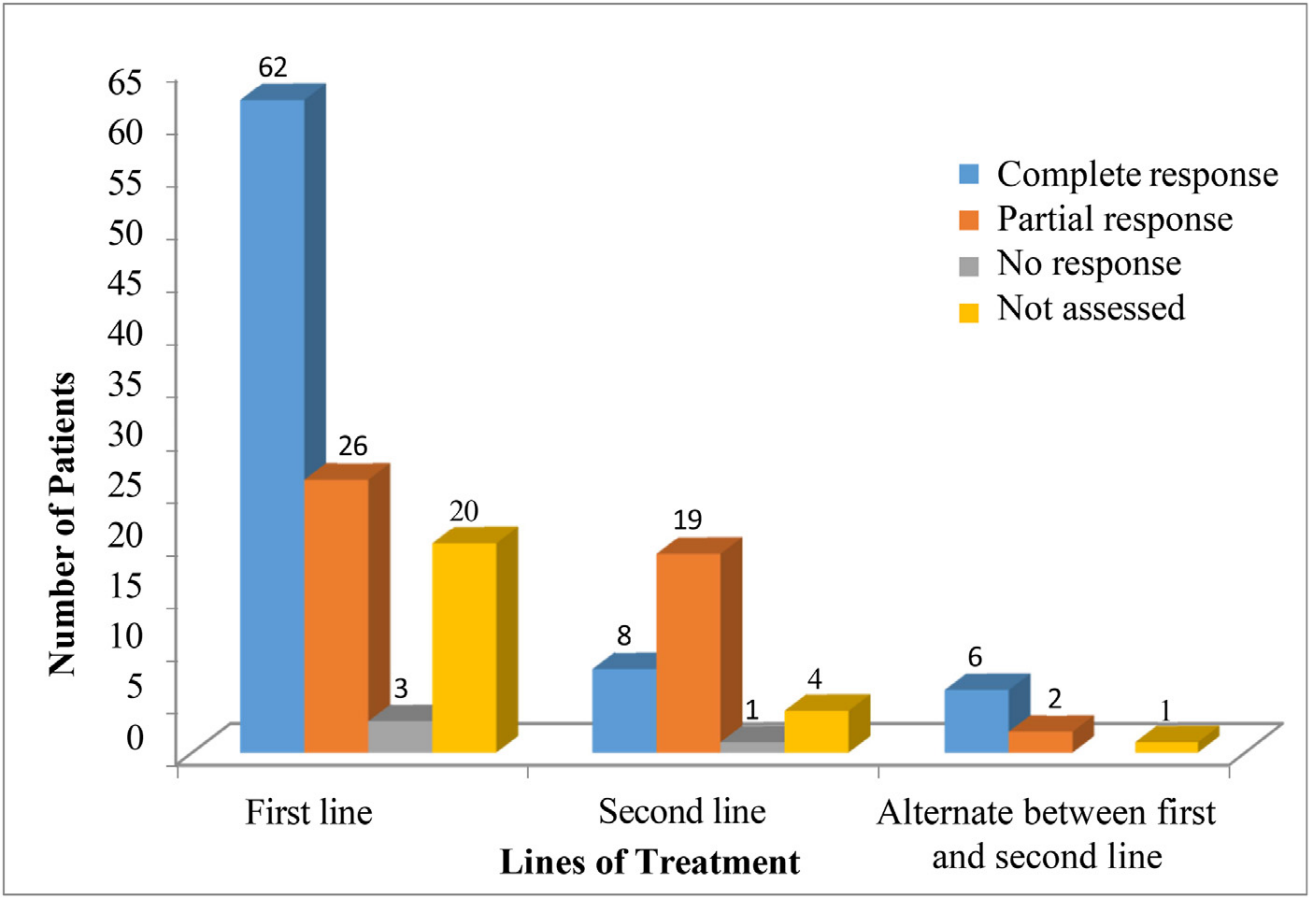
Treatment responses of first and second line regimens of the INCTR-03-06 protocol.

Of the 90.1% (111/123) of children who received first-line chemotherapy, 55.9% (62/111) and 23.4% (26/111) achieved a CR and PR, respectively, while 2.7% (3/111) had NR. Twenty patients (18.0%) could not be assessed for response following first-line treatment; of these, 9.9% (11/111) died and 8.1% (9/111) were lost to follow-up (Figure 2). Subsequently, all patients with PR or NR, along with three patients referred from an external cancer center, were switched to second-line chemotherapy. In total, 32 (28.8%) of the patients transitioned to second-line regimens, 28.5% (8/32) and 59.4% (19/32) of whom achieved CR and PR, respectively and were discharged home for palliation. Additionally, of the nine children with bulky abdominal disease who were kept on a hybrid regimen of first and second lines, six (66.7%) children achieved CR.

#### Predictors of mortality among children with Burkitt lymphoma

In univariate analysis, the risk of death was three times higher in HIV infected children compared to HIV negative children (unadjusted Hazard Ratio [uHR] = 3.07; 95% CI: 1.19–7.59; p = 0.02). Additionally, children in advanced disease risk groups (C and D) had a 2.3-fold increased risk of mortality compared to those in risk group B (uHR = 2.34; 95% CI: 1.09–5.05; p = 0.03). Moreover, it was observed that children with increased LDH levels (>500 U/L) had increased risk of death compared to their counterparts (uHR = 1.58; 95% CI: 1.28–3.20; p = 0.04). Multivariate Cox regression analysis was performed, adjusting for CSF involvement, risk stratification, HIV status, and serum LDH levels. HIV-positive status and elevated LDH (>500 U/L) remained independently associated with an increased risk of mortality. Specifically, HIV-positive patients had a fivefold higher risk of death compared to HIV-negative patients (aHR = 5.1; 95% CI: 1.38–19.0; p = 0.02). Furthermore, patients with LDH levels >500 U/L faced a significantly higher risk of mortality than those with LDH levels ≤500 U/L (aHR = 2.50; 95% CI: 1.14–14.71; p = 0.03). Although children in risk groups C and D exhibited higher mortality rates compared to those in risk group B, this difference did not reach statistical significance (aHR = 1.83; 95% CI: 0.42–8.08; p = 0.42) (Table 3).

**Table 3:** Cox proportional hazards analysis for predictors of mortality of children with Burkitt lymphoma (n = 123).

	Patient status		Univariate analysis		Multivariate analysis	
Variable	Dead	Alive	uHR (95% CI	p-value	aHR (95% CI)	p-value
Sex						
Female	15 (31.9)	32 (68.1)	1.0			
Male	26 (34.2)	50 (65.8)	0.93 (0.60- 1.76)	0.83		
Age (years)						
<10	26 (63.4)	15 (36.6)	1.0			
≥10	15 (18.3)	67 (81.3)	0.56 (0.18–1.71)	0.31		
Tumor sites						
Jaw	9 (29.0)	22 (71.0)	1.0			
Abdomen	17 (35.4)	31 (64.6)	1.29 (0.58–2.88)	0.53		
Others	6 (31.6)	13 (68.4)	1.13 (0.74–5.11)	0.62		
CSF test						
Negative	12 (20.7)	46 (79.3)	1.0		1.0	
Positive	14 (35.9)	25(64.1)	1.83 (0.86–3.89)	0.12	0.64 (0.15- 2.85)	0.56
Bone marrow test						
Negative	14 (25.5)	41 (74.5)	1.0			
Positive	4 (33.3)	8 (66.7)	1.29 (0.43- 0.39)	0.65		
Tumor staging						
Stage I and II	10 (31.2)	22 (68.8)	1.0		1.0	
Stage III and IV	31 (47.0)	35 (53.0)	2.34 (1.09–5.05)	0.03	1.83 (0.42- 8.08)	0.42
Risk group						
Low-risk	3 (25.0)	9 (75.0)	1.0			
High-risk	37 (43.0)	49 (57.0)	1.27 (1.35–8.13)	0.49		
HIV status						
HIV-	36 (31.3)	79 (68.7)	1.0		1.0	
HIV+	5 (62.5)	3 (37.5)	3.07 (1.19–7.95)	0.02	5.12 (1.38–19.0)	0.02
LDH (U/L)						
≤500	10 (25.0)	30 (75.0)	1.0		1.0	
>500	31 (37.3)	52 (62.7)	1.58 (1.28–3.20)	0.04	2.50 (1.14- 14.71)	0.03
Hb (g//L)						
≥115	6 (27.3)	16 (72.7)	1.0			
<115	35 (34.7)	66 (65.3)	1.32 (0.71–2.47)	0.3		

uHR: unadjusted Hazards ratio; aHR: adjusted Hazard ratio; CI: confidence interval; CSF: cerebrospinal fluid; HIV: human immunodeficiency virus; LDH: lactate dehydrogenase; Hb: haemoglobin.

#### Survival rates of children with Burkitt lymphoma

At the end of the study period, 49 (39.8%) of the patients were in clinical remission. However, 33 patients (26.8%) were lost to follow-up. Among these, 13 (39.4%) abandoned treatment before response assessment, while the remaining 20 were unreachable by telephone: 11 (33.3%) had inactive numbers and nine (27.3%) did not respond to repeated call attempts. A total of 82 patients (66.7%) were censored in the survival analysis. Of these, 33 (40.2%) were censored due to loss to follow-up, while 49 (59.8%) were censored because they had not experienced the event of interest (death) by the end of the study period. Death was confirmed for 41 (33.3%) of the children analyzed in the study, with 11 (26.8%) deaths attributed to disease and treatment-related complications during treatment and the other 30 (73.2%) occurring at home.

The mean survival time from the initiation of treatment to 18 months was 13 months (95% CI: 11.7–14.3), with an estimated 18-month OS rate of 54.0% (Figure 4). The estimated OS rate among children with elevated LDH (>500 U/L) was shorter than that of children with LDH levels of ≤500 U/L (44.9% versus 72.4% - Log rank: p < 0.001) (Figure 5). Also, children who were HIV positive had reduced OS compared to children who were HIV negative (29.2% versus 61.7% - Log rank: p = 0.01) (Figure 6). Although the OS rate for children with jaw disease was higher compared to children with abdominal disease (70.5% versus 56.0%), the difference was not statistically significant (Log-rank: p = 0.43) (Figure 7).

**Figure 3:**
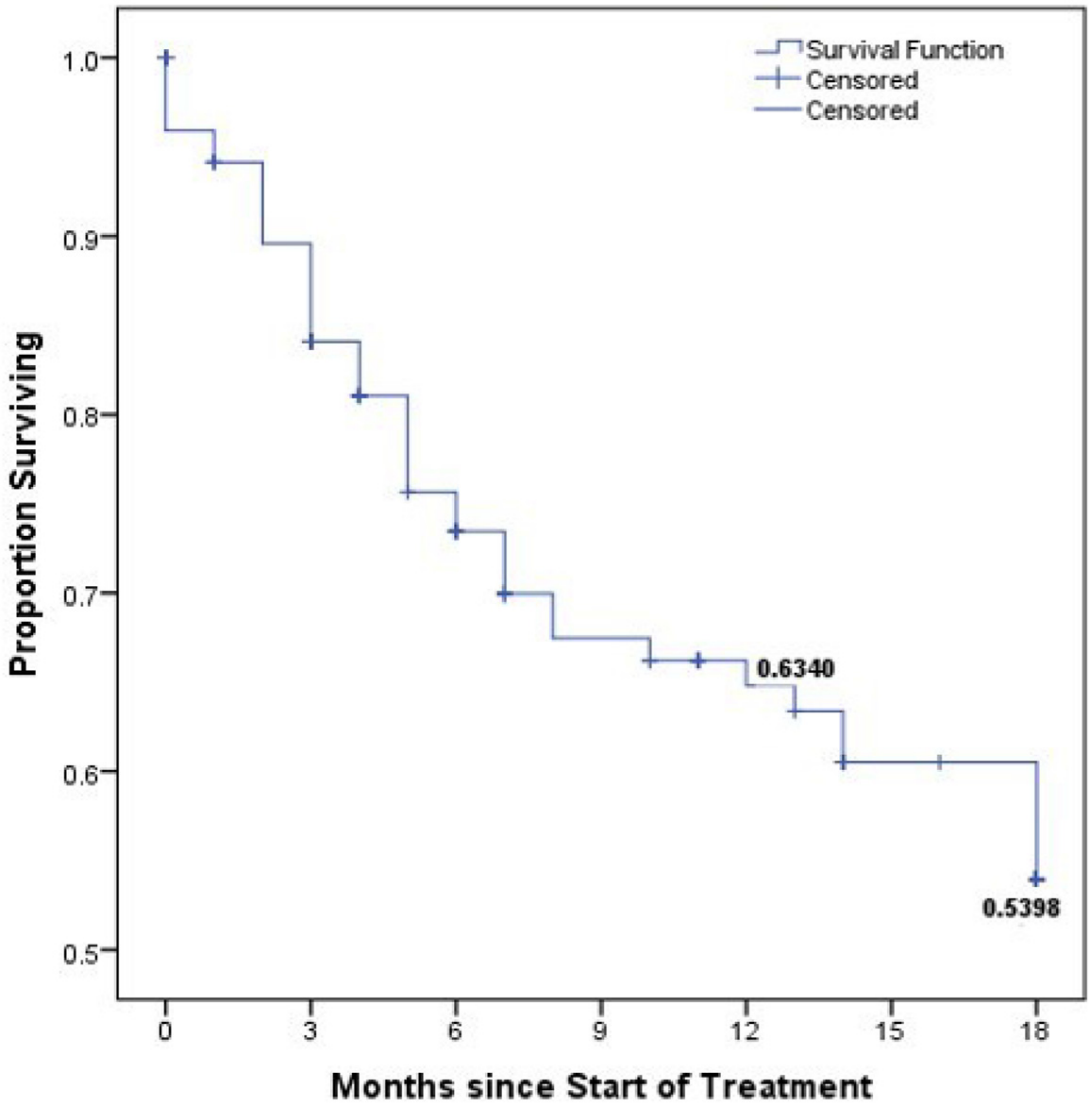
Kaplan-Meier curve of the overall survival of patients treated for Burkitt lymphoma.

**Figure 4:**
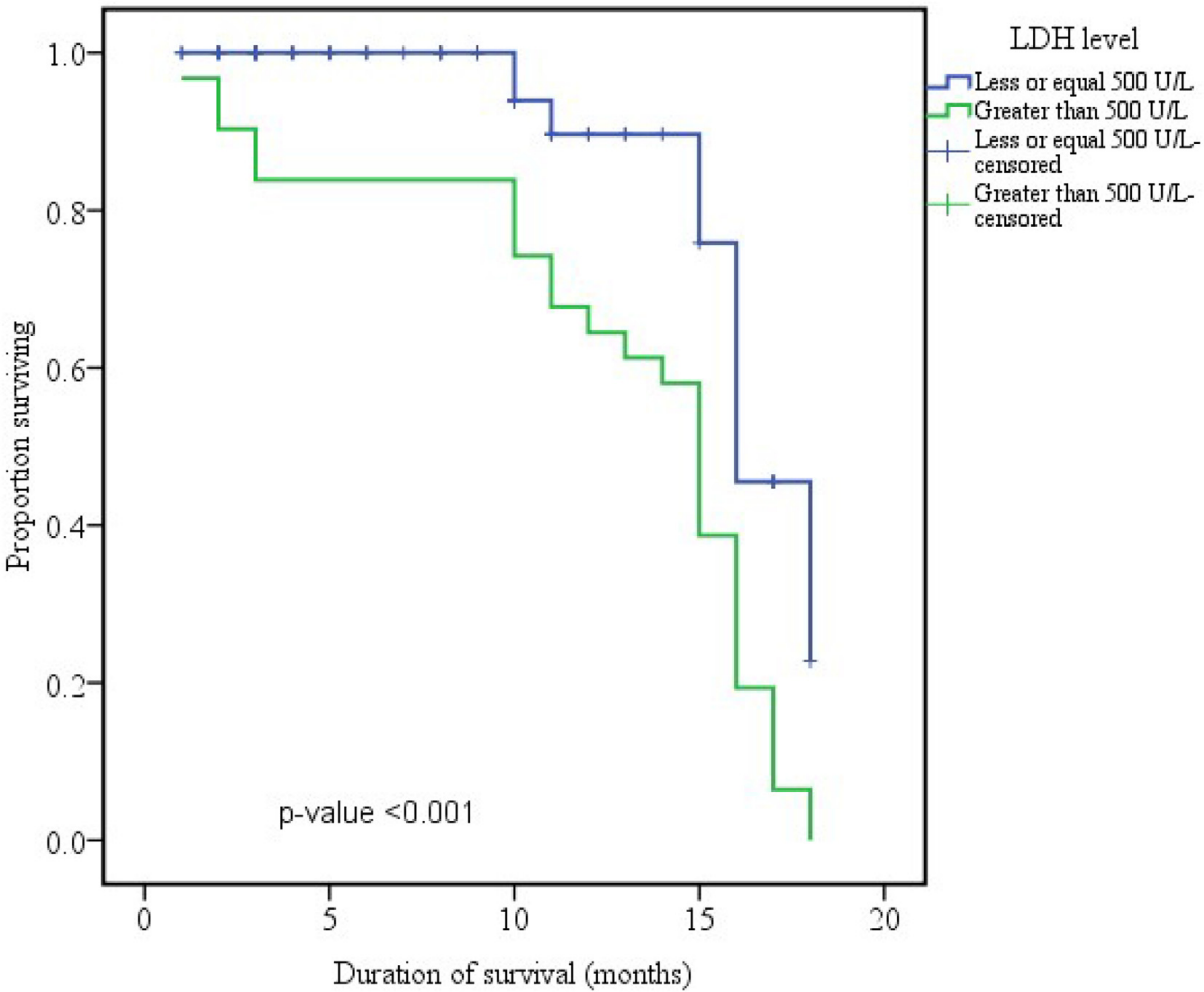
Kaplan-Meier survival analysis of patients stratified by serum lactate dehydrogenase (LDH) levels.

**Figure 5:**
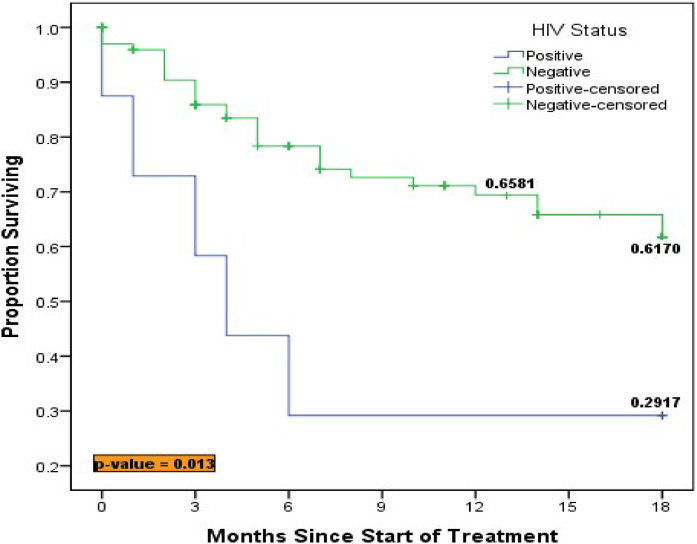
Kaplan-Meier curves comparing overall survival of patients stratified by HIV status.

**Figure 6:**
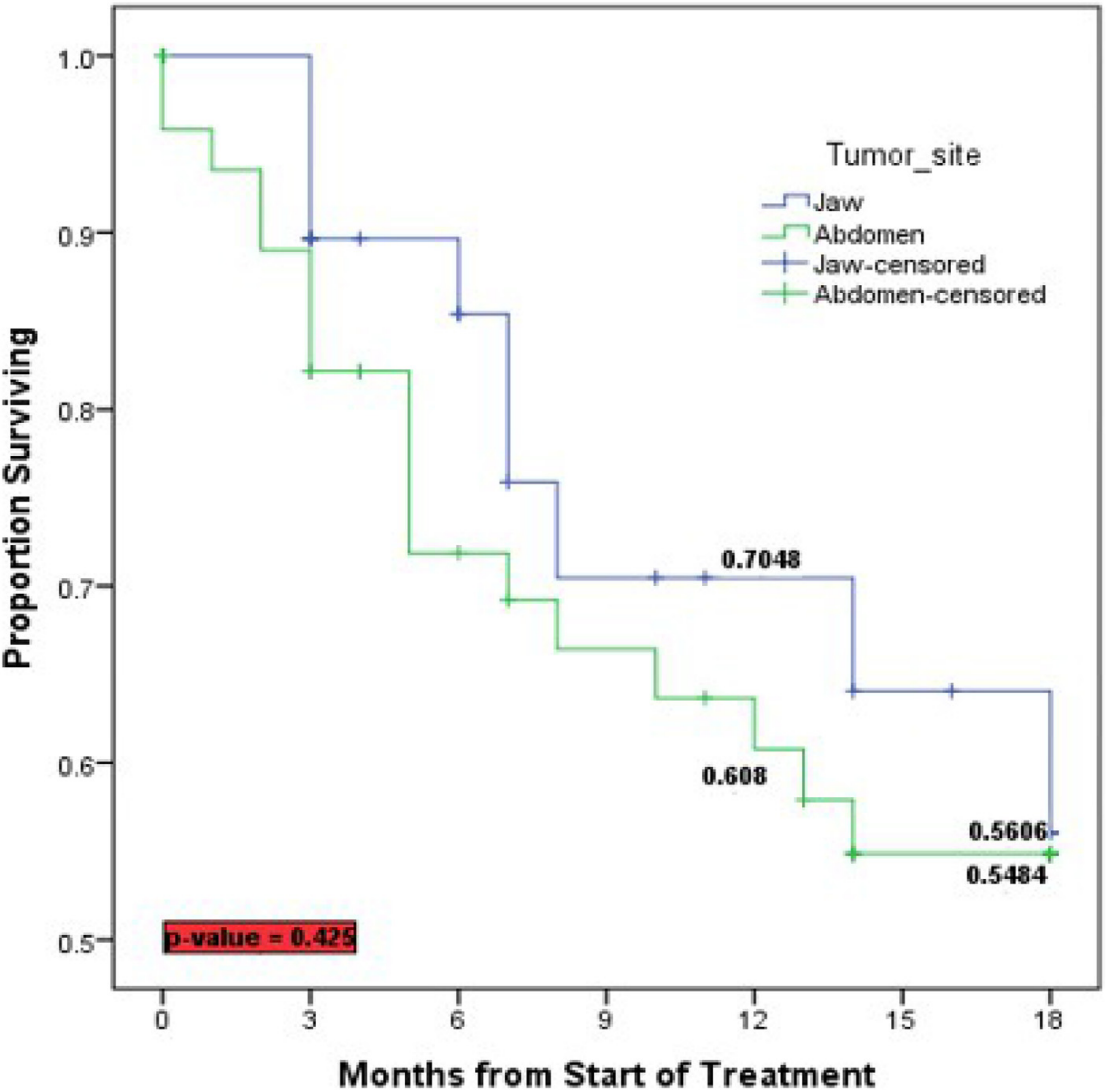
Kaplan-Meier curves comparing overall survival of patients stratified by tumor site.

## Discussion

This study sought to assess the clinical profile and predictors of mortality of children with BL who were treated at a national hospital in a resource-constrained setting in the southern region of sub-Sahara African. The key findings of this study demonstrate a moderate OS rate. Furthermore, HIV-positive status and elevated LDH levels were identified as significant independent predictors of mortality in this patient population.

In the present study, abdominal manifestations were the most common clinical presentation of BL, followed by jaw involvement. This aligns with findings from studies conducted in Mozambique and Uganda [7,22]. Similarly, research from Malawi, Northern Uganda, Cameroon, and Tanzania has reported a clear abdominal predilection in the clinical presentation of BL [10,23, 24, 25]. The rising proportion of patients presenting with abdominal disease may be partially attributed to advancements in diagnostic modalities. The widespread availability of abdominal ultrasound, CT, and MRI in equatorial Africa has enhanced the detection of intra-abdominal involvement, which is in contrast to historical trends where jaw involvement was the predominant clinical feature [26].

Regarding treatment responses in this study, over half of the patients achieved CR on first-line chemotherapy of the INCTR protocol. The CR rate in this study (55.9%) was lower than those reported in other regional cohorts; for instance, Ngoma et al. [19], Park et al. [27], and Stanley et al. [10] observed CR rates of 75.8%, 76.3%, and 88%, respectively. However, a slightly lower proportion (44.5%) was reported in Uganda [2]. Discrepancies in CR rates across these studies may be attributed to variations in chemotherapy protocols, as well as differences in clinical staging, risk stratification, and the performance status of the patients at presentation. Despite marked variations in CR rates following first-line chemotherapy across these studies, the early mortality rate in this cohort did not differ significantly from rates reported in previous research. Furthermore, the proportion of patients achieving CR after transitioning to second-line regimens was low, a finding consistent with outcomes reported in other studies [10,19,27].

In this study, the 18-month OS was 54.0%; this is slightly lower than the 1-year and 2-year OS rates of 67.0% and 62.0%, respectively that were reported by Ngoma et al. in Tanzania in 2012 [19]. In a similar cohort from Malawi, the 18-month OS rate was reported to be 54% [14], which is consistent with the findings observed in the present study. In Uganda, McGoldrick et al. reported a 4-year OS rate of 44%, with 88% of their cohort treated using the COM regimen [28]. Furthermore, in the study of Fahad et al. [29] in the United States, the 5-year OS was 90.4%, that is 54.0% higher than the 18-month OS rate found in the present study. Amani et al. also reported a 5-year OS rate of 56% among children treated with BL in Saudi Arabia, 2% higher than the 18-month OS rate in the present study.

Several factors may explain the discrepancies in OS observed across these studies, most notably the wide variation in chemotherapy intensity across sub-Saharan Africa. For instance, the high-intensity LMB protocol used in South Africa yielded a superior OS rate of 64.7%. In contrast, the JOOTRH protocol utilized in Kenya resulted in a 1-year OS of 45%, while the lower-intensity CHOP regimen, previously used in Malawi for non-Hodgkin lymphoma including BL, demonstrated a lower 1-year OS rate of 29.0% [10,30,31]. Since the implementation of the INCTR-03-06 protocol, a standardized, non-trial treatment strategy primarily based on the COM regimen, there has been a significant improvement in the OS of patients with BL [19,32]. The difference in the risk groups as well as tumor stages of patients in the studies may help to explain the discrepancies in the OS rates. Studies have shown that, patients who are classified into high risk groups (C and D), generally have a poorer clinical outcome compared to patients who are in low risk groups (A and B) [33,34].

Studies from the sub-Sahara African region have shown several factors that can influence the clinical outcome of the patients. In the present study, the clinical stage of the disease at the time of diagnosis, was strongly associated with prolonged survival. Children with advanced disease (risk groups C and D) were at increased risk of dying compared to children who were in risk group B. This finding agrees with the observation in the studies carried out in the United States, South Africa, and Ghana [29,31,35]. Children with advanced stage BL present in fragile states with a multitude of complications and huge tumor cell burden, which increases their risk of early mortality [36]. Further, most children with cancer in low- and middle-income countries present late to cancer treatment centers, this contributes significantly to advancing the disease before initiation of primary treatment [37]. HIV infection was another predictor of mortality in the current study. HIV-positive patients had shorter survival compared to HIV-negative children. This observation aligns with the findings of Orem et al. in Uganda, who reported that HIV-positive status was significantly associated with inferior OS [38]. Another similar finding was reported in the study of Stefan et al. in South Africa, which found that the mortality rate was significantly higher in the HIV-positive group compared to the HIV-negative group (73% versus 23.0%) [31]. HIV infection remains a significant negative prognostic factor, despite the concurrent administration of antiretroviral therapy (ART) and cytostatic treatment [31].

LDH, an enzyme present in most body cells, is released into the bloodstream when cells are damaged or destroyed [39]. LDH is a known biomarker of tumor burden as well as of cell turnover [40, 41, 42], and therefore high levels of LDH in cancer patients suggest a larger amount of cancerous tissue and rapid cell death [43]. In this study, patients who had an elevated level of LDH (≥500 U/L) had a higher mortality rate. This finding is in agreement with the findings of the studies by Cairo et al. [44], Buckle et al. [10], and Galleze et al. [41].

In the present cohort, children with jaw involvement exhibited superior OS compared to those with abdominal BL, although the difference did not reach statistical significance. This trend aligns with previous studies that have consistently demonstrated more favorable survival outcomes in patients with jaw presentations relative to those with intrathoracic or abdominal involvement. For example, Nkurumah et al. reported poorer prognosis of children with BL involving the abdomen than those having jaw involvement [45]. Two review articles, one by Linch and the other by Crombie & LaCasce, explained that jaw involvement carries a better prognosis than intra-thoracic or intra-abdominal involvement [46,47]. Several factors may explain this prognostic advantage. First, jaw involvement is more characteristic of pediatric BL than adult cases, and children generally achieve superior clinical outcomes compared to their adult counterparts [48, 49, 50]. Additionally, jaw-associated BL often carries a more favorable prognosis due to its typically localized nature at presentation, which contrasts with the more disseminated disease often seen in other primary sites. Furthermore, jaw lesions are more accessible for biopsy or primary surgical resection, which, when combined with chemotherapy, may improve patient outcomes compared to those with deep-seated visceral disease [51,52].

Other factors such as age, sex, and hemoglobin level did not affect the OS of the patients in this study. This is contrary to the observations by Stanley et al. which showed that mortality was associated with being aged >9 years, and being female [10].

### Limitations of this study

The retrospective nature of the study contributed to recall bias in one way or another as not all caretakers could recall the exact time of event (death or relapse). The sample size, which is relatively small, affected the power of the study. Because data were collected from a single institution, the findings may not be generalizable to other settings across the region. Additionally, potential biases in predictor selection, and lack of external validation were other factors which affected the findings of this study.

## Conclusion

In this study, abdominal involvement was the most frequent clinical presentation, and approximately half of the patients achieved CR following first-line chemotherapy. The 18-month OS rate remained low, with HIV infection and elevated LDH levels identified as key predictors of early mortality. Consequently, thorough pretreatment evaluation of pediatric BL should mandate HIV screening alongside bone marrow and CSF investigations, as these factors are critical determinants of patient survival.

## Ethical clearance and consent to participate

Ethical clearance was obtained from the Research and Publication Committee of MUHAS. Permission to conduct the study in the pediatric department was obtained from the Research Ethics Committee of the MNH. All patients’ information was anonymized using unique codes for the purpose of confidentiality. Verbal consent was obtained from patents or caregivers during the interview and they were informed of their rights to participate including withdrawing at any point from the interview.

## Funding

No funding was received for the research, authorship, or publication of this article.

## Patient consent statement

The caregivers of the study subjects gave their informed consent.

## Authors’ contribution

AAS, AIN, NSM, SJ: study designing, conceptualization, supervision, and data curation. AAS, JJY, AIN: methodology, statistical analysis, and interpretation, writing the first draft of the manuscript. All authors reviewed the final version of the manuscript critically and agree to take responsibility for the intellectual part of it.

## Data availability statement

The data that support the findings of this study are available from the corresponding author upon reasonable request.

## Conflicts of interest

None of the authors have competing interests to disclose regarding this work.
